# Implementing health policies in Australian junior sports clubs: an RCT

**DOI:** 10.1186/s12889-019-6873-3

**Published:** 2019-05-14

**Authors:** Tara Clinton-McHarg, Sharleen Gonzalez, Sharin Milner, Shauna Sherker, Melanie Kingsland, Christophe Lecathelinais, Alix Hall, Chris Doran, John Wiggers, Luke Wolfenden

**Affiliations:** 1 Priority Research Centre for Health Behaviour (PRCHB), School of Medicine and Public Health, The University of Newcastle, Callaghan, NSW 2308 Australia; 2 Hunter Medical Research Institute, New Lambton Heights, NSW 2305 Australia; 3 Alcohol and Drug Foundation, Melbourne, VIC 3051 Australia; 4 Hunter New England Population Health, Locked Bag 10, Wallsend, NSW 2287 Australia; 5 Centre for Indigenous Health Equity Research, Central Queensland University, Brisbane, QLD 4000 Australia

**Keywords:** Sporting clubs, Randomised controlled trial, Implementation, Prevention, Risk factors

## Abstract

**Background:**

This pilot study aimed to test the potential effectiveness and acceptability of an intervention to support the implementation of 16 recommended policies and practices to improve the health promotion environment of junior sporting clubs. Reported child exposure to health promoting practices at clubs was also assessed.

**Methods:**

A cluster randomised trial was conducted with eight football leagues. Fourty-one junior football clubs belonging to four leagues in the intervention group received support (e.g. physical resources, recognition and rewards, systems and prompts) to implement 16 policies and practices that targeted child exposure to alcohol, tobacco, healthy food and beverages, and participation in physical activity. Thirty-eight clubs belonging to the four control group leagues did not receive the implementation intervention. Study outcomes were assessed via telephone interviews with nominated club representatives and parents of junior players. Between group differences in the mean number of policies and practices implemented at the club level at follow-up were examined using a multiple linear regression model.

**Results:**

While the intervention was found to be acceptable, there was no significant difference between the mean number of practices and policies reported to be implemented by intervention and control clubs at post-intervention (Estimate − 0.05; 95% CI -0.91, 0.80; *p* = 0.90). There was also no significant difference in the proportion of children reported to be exposed to: alcohol (OR 1.16; 95% CI 0.41, 3.28; *p* = 0.78); tobacco (OR 0.97; CI 0.45, 2.10; *p* = 0.94); healthy food purchases (OR 0.49; CI 0.11, 2.27; *p* = 0.35); healthy drink purchases (OR 1.48; CI 0.72, 3.05; *p* = 0.27); or participation in physical activity (OR 0.76; CI 0.14, 4.08; *p* = 0.74).

**Conclusions:**

Support strategies that better address barriers to the implementation of health promotion interventions in junior sports clubs are required.

**Trial registration:**

Retrospectively registered with the Australian New Zealand Clinical Trials Registry (ACTRN12617001044314).

**Electronic supplementary material:**

The online version of this article (10.1186/s12889-019-6873-3) contains supplementary material, which is available to authorized users.

## Background

Tobacco smoking, high levels of alcohol consumption, poor dietary behaviour and physical inactivity are risk factors for the development of cardiovascular disease, cancer, type 2 diabetes, and other chronic diseases in adulthood [1]. If these behaviours and risks are established in childhood and adolescence, they are more likely to continue in later life [2–4]. Recent studies in the United Kingdom and Australia have shown that only 1 to 10% of children and adolescents under the age of 18 meet the recommended daily intake of vegetables [5, 6], and less than 25% are physically active for more than the recommended 60 min per day [7, 8]. Similarly, by the age of 17 years, up to 19% of adolescents are reported to be drinking at levels that could result in short-term harm [9], and in England 7% of 15 year olds reported they were regular smokers, while 8% reported they were occasional smokers [10] .

In Australia it is estimated that 60% of children aged 5–14 years participate in organised sporting activities, such as those offered by community sporting clubs outside of school hours [11]. While sporting clubs provide important community infrastructure to support child physical activity [12], enjoyment of sport and desire for future participation can be limited by poor sideline behaviour from parents (e.g. negative vocalisation) [13]. In addition, other health promoting practices are often poorly implemented in these settings. For example, an Australian study reported that over 90% of sporting club canteens sold sugar-sweetened drinks, confectionery, pastries and salty snacks, while less than 34% sold products containing fruit or vegetables [14]. Children’s exposure to excessive alcohol consumption and tobacco is also reported to be common at sporting events [15], and is potentially facilitated by the poor implementation of anti-tobacco polices or alcohol management practices at sporting venues [16]. Therefore, interventions in community sporting clubs have considerable scope to improve a variety of child health behaviours [12, 17].

Despite a limited number of trials in this setting, interventions aimed to assist junior community sporting clubs to implement policies and practices that support health behaviours in young people have been found to be beneficial [18, 19]. For example, a randomised trial in Australia reported that an intervention targeting coaching practices lead to an improvement in adolescent girls’ physical activity intensity during sport [19, 20]. In Canada, a non-randomised trial reported an increase in the availability of healthy foods for sale at sporting clubs, as well as improved purchasing of these products (predominately for children) [18]. These results are consistent with findings from consensus processes undertaken with experts and sporting club stakeholders that have recommended a range of policies and practices that sporting clubs should adopt to promote child health [21]. Recommendations included: creating smoke free environments; restricting alcohol sales; increasing the availability of healthy foods for sale at club canteens; and social inclusion policies such as ensuring equal time on the field for junior players during games [21].

While high level evidence of the benefit of health promoting policies and practices in junior sporting clubs is currently lacking, systematic reviews [16] have identified a small number of controlled trials that have observed improvements in the implementation of practices to reduce excessive alcohol consumption [22, 23] and improve the purchase of healthier foods by adults attending community sporting club fixtures [24]. Such recommended policies and practices need to be implemented if they are to improve the health promoting environment at community sports clubs and benefit children.

In this context, the aim of this pilot study was to assess the potential effectiveness and acceptability of a multi-component intervention to support the implementation of a range of recommended policies and practices targeting alcohol and tobacco use, healthy food and beverage provision, participation in sport, and member conduct. The potential impact of the implementation of such policies and practices on child exposure to: alcohol and tobacco use; healthy food and beverage purchasing from the club canteen; provision of healthy snacks by parents; equal participation in training and games; and a safe playing environment were also assessed.

## Methods

### Design and setting

This was a cluster randomised-controlled trial, with football leagues as the unit of randomisation. Junior football clubs in football leagues from metropolitan and regional areas in the states of Victoria and New South Wales (NSW), Australia were included. A full description of the trial protocol has been published elsewhere [25]. The study adheres to CONSORT guidelines.

### Participants

#### Football leagues

All Australian Football Leagues (AFL) in Victoria, and all Rugby Leagues and Country Rugby Leagues in NSW constituted the sampling frame for the study. Football leagues are the overarching organisations to which individual football clubs belong. To be eligible, football leagues needed to: 1) be a community-level (non-professional) league; and 2) have 10 or more junior football clubs within the league who had Level 3 accreditation with a national health promotion program (*Good Sports)* [26]. The *Good Sports* program supports sporting clubs to implement alcohol management practices (if alcohol is sold) using a three-level accreditation process, with Level 3 being the highest accreditation status [27]. The alcohol management practices targeted in this intervention were in addition to those required in the *Good Sports* program. Football leagues who were already participating in other research trials were excluded.

#### Junior football clubs

Individual junior football clubs were eligible to participate in the trial if they belonged to an eligible league and had more than 40 registered junior players. From each eligible junior club, a club representative (e.g. president, secretary, committee member) was nominated to complete a telephone interview on behalf of their club. Club representatives needed to be 18 years or older and speak English.

#### Parents of junior players

Parents of junior players at participating clubs were eligible to take part in a telephone interview if they were 18 years or older and spoke English.

### Recruitment procedures

#### Football leagues

A member of the research team met with representatives from all football leagues who met the eligibility criteria, to inform them about the research trial and invite their league to participate. Following this meeting, league representatives provided written consent for their league to participate.

#### Junior football clubs

Once an eligible league consented to participate, a member of the research team attended the next league meeting to inform the representatives of all junior clubs in the league about the research trial and invite their participation. Following this meeting, a nominated club representative was emailed an information statement and a consent form.

#### Parents of junior players

Representatives from participating junior clubs were emailed electronic copies of study information statements and were asked to distribute them to up to 20 parents (or carers) of junior players at the club (either as electronic or hard copies). Parents who were willing to participate in the telephone interviews provided consent for the club representative to forward their name and telephone contact details to the research team. The eligibility of the parents, and their verbal consent to participate in both the baseline and post-intervention telephone interviews (cohort design), were confirmed at the commencement of both interviews.

### Random allocation

**Fig. 1:**
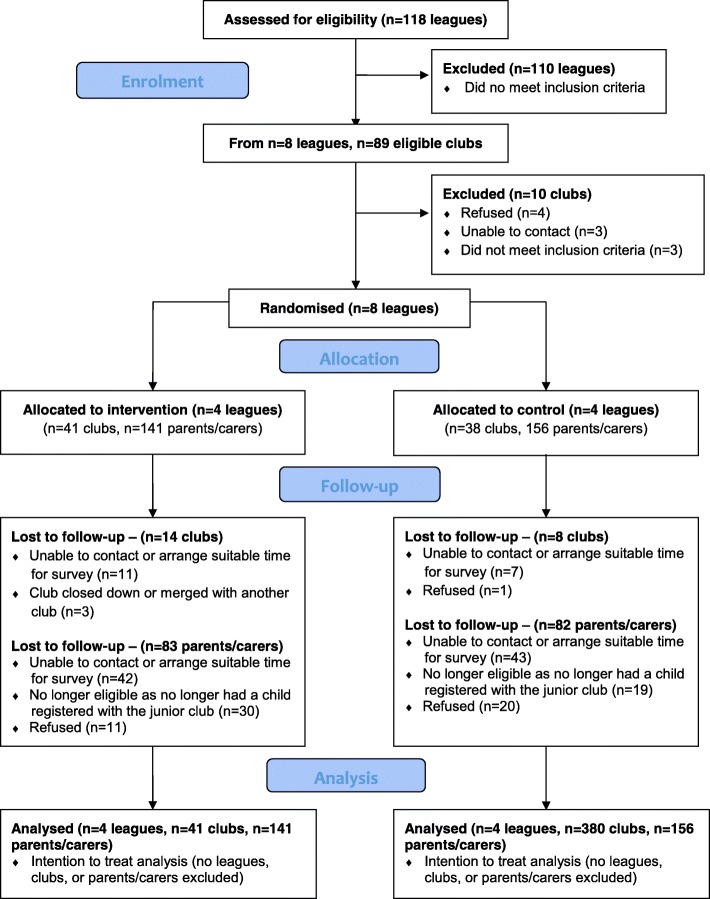
Consort flow chart showing the progress of participants through the trial

Consenting state leagues were randomly allocated to either the intervention group, or control group, in a 1:1 ratio by an independent statistician using a computer-generated randomisation sequence. Leagues were stratified by football code (Australian Football League [AFL] or Rugby League) and geographic location (Victoria or New South Wales [NSW]) (Fig. 1).

### Multi-component intervention including implementation support

The intervention ran for one winter football season (approximately 6 months) which in Australia begins around March/April and usually ends in August/September.

#### Intervention content

An expert advisory group consisting of experienced drug and alcohol researchers, health promotion practitioners, and behavioural and implementation scientists developed the intervention. The intervention content consisted of 16 policies and practices (refer to Additional file 1) that junior football clubs in the intervention group were required to implement. The 16 policies and practices were chosen as they were supported by evidence from previous trials in sporting club settings [22–24] or recommended following consensus processes with health promotion and sports management experts [21]. The 16 policies and practices were as follows:

1. Alcohol is not available or consumed during junior competition
2. Alcohol is not available or consumed at junior events or presentations
3. Alcohol is not present in the change rooms when players under 18 years are present
4. Alcohol manufacturers, wholesalers, retailers or other businesses whose core function is to sell alcohol are not promoted or advertised by the club on any junior apparel
5. Alcohol is not used for prizes, rewards or for fundraising
6. The club is compliant with the relevant state tobacco legislation
7. The club promotes all junior events as smoke free
8. Water is promoted as the drink of choice for junior players
9. Multiple healthy food and beverage (eg, fruit, vegetables and non-sugar-sweetened drink) options are available at the canteen or barbecue
10. The purchase of healthy choices at the canteen or barbecue are promoted by ensuring healthy food and beverage options are displayed prominently
11. The club encourages parents to provide healthy snacks (eg, fruit and water) for junior players
12. The club conducts at least one recruitment activity prior to the beginning of the winter sporting season to attract new junior players and retain current players
13. The club has a Participation policy that it communicates to members, coaches, officials and volunteers to ensure junior players are provided with equal opportunities for participation at both training and during games
14. The club has a Code of Conduct policy that it communicates to all members and ensures member agreement is recorded
15. The club has a Spectator Behaviour policy that is promoted and clearly visible at the club
16. The club has a written Good Sports Junior policy, which outlines the club’s practices with regards to alcohol consumption, tobacco use, healthy eating and physical activity at junior competitions and events

#### Intervention delivery

Staff who were already experienced in delivering the national *Good Sports* program supported intervention clubs throughout the intervention period with: three support phone calls; three tailored support emails; and five automated emails. Automated emails were sent every month, while support phone calls and tailored support emails were alternated each month.

##### Three support phone calls

The first phone call was used to review the club’s current policies and practices, and help clubs to identify the intervention practices/policies they still needed to implement. This was done using a standardized, electronic action plan that support staff filled in while talking to the club representative. Following this phone call, clubs were mailed a resource pack and received a list of actions that individual clubs had agreed to complete over the phone. The following two phone calls were used to monitor how the clubs were progressing.

##### Three tailored support emails

Support staff sent three emails to the club representative encouraging them to implement relevant polices or practices in the coming month. These emails were tailored to the areas of specific need identified for each club according to their action plan.

##### Five automated emails

Monthly, automated support emails were also sent to club representatives throughout the sporting season. The automated emails were generic and focused on the themes of physical activity, healthy eating, alcohol consumption, smoking and member conduct. The emails included information that clubs could use on their website or social media page to communicate changes to club members.

#### Implementation support strategies

The NSW Health Capacity Building Framework [28] was used to identify potential areas where support could be provided to clubs, to assist them to implement the 16 policies and practices. Potential barriers to implementation were identified in previous trials with sporting clubs [17, 29] and by consultation with personnel experienced in working with sporting clubs. Strategies to address the identified barriers were developed in three key areas of the NSW Health Capacity Building Framework: 1) Organisational Development; 2) Workforce Development; and 3) Resource Allocation.

##### Organisational development

- Leadership support – State football Leagues endorsed the research trial via an email to their clubs, and encouraged club participation.
- Policies and procedures – Club representatives received hardcopy and electronic policy templates to assist their clubs develop health promotion policies. For example, to increase the physical activity of existing members, templates were supplied to support clubs develop policies regarding equal game time participation for all players. Club representatives also received a procedural document, which outlined possible recruitment strategies that the club could use to attract new junior players and retain existing players.
- Recognition and reward systems – Club representatives received recognition of progress towards implementing all 16 policies and practices during regular phone calls from support staff. Clubs who implemented all policies and practices received a certificate of accreditation and a digital asset pack. The digital asset pack contained electronic templates to promote their achievement on their club website or social media page, or to send to local media outlets.
- Information systems – An online data management system was used by support staff to monitor each clubs progress towards the implementation of the 16 policies and practices. The online system allowed support staff to provide real-time feedback to clubs, by generating action plans that identified which intervention criteria still needed to be completed. Clubs were encouraged to upload evidence of their implementation on to the online system (i.e. photos, copies of policies) to show the support officers that the practice had been implemented. The support staff could then update the club’s action plan accordingly.
- Systems and prompts – Clubs received email reminders from support staff to prompt the implementation of relevant policies and practices, as well as automated, ‘themed’ emails addressing physical activity, healthy eating, alcohol consumption, smoking, and member conduct.
- Promoting culture – Clubs were encouraged to select rounds of the junior competition, or a junior event, to focus on promoting the 16 policies and practices to members (e.g. the alcohol awareness round, or the healthy eating round).

##### Workforce development

- Developing skills and knowledge - Clubs received an alcohol management toolkit, which provided them with step-by step instructions on strategies they could use to implement the five alcohol practices. This included examples of ways that clubs could: communicate practices effectively with members; identify members who could be responsible for monitoring and enforcing practices; re-negotiate with alcohol promoters and sponsors to change advertising agreements; deal with member non-compliance; and establish processes for reporting non-compliance.

##### Resource allocation

- Human resources – Support staff were allocated to intervention clubs to help them implement the intervention. This included monitoring and providing feedback on the implementation of practices. Assistance was also provided via regular phone and email contact with individual club representatives (once per month) during the winter season to maintain support.
- Physical resources – All intervention clubs received a hard copy resource kit at the beginning of the intervention period. The kit included: posters promoting alcohol-free junior competitions; alcohol-free change room signs; a list of alternate prizes to alcohol for fundraisers, raffles or gifts to coaches; smoke-free posters; a canteen whiteboard to promote healthy food and beverage options prominently; a safe food handling poster; letter templates for clubs to send to parents to encourage them to provide healthy snacks for juniors; and a playing environment sign with the *Good Sports* Code of Conduct prominently displayed, and other similar signs, posters and letter templates. Electronic versions of these resources were also provided throughout the season via email. A lead sporting figure from each participating football code appeared in resource materials.

### Control group clubs

Control clubs did not receive any implementation support or resources during the intervention period from the research team.

### Data collection and measures

#### Primary outcome

The primary outcome of the trial was change in the mean number of policies and practices (out of 16) implemented by junior sporting clubs. Outcome data were collected via computer-assisted telephone interviews (CATIs) with the nominated club representatives at baseline (July–September 2016) and post-intervention (August–November 2017). The survey items were developed by the research team to align with the 16 policies and practices, and internally piloted before use. The telephone interview took approximately 30 min to complete.

#### Secondary outcomes

A secondary outcome for the trial was the proportion of clubs that implemented each of the 16 policies and practices (refer to Additional file 1). These data were collected from the club representatives during the baseline and post-intervention CATIs described above.

Other secondary outcomes were:

##### Child exposure to alcohol

Parents of junior players were asked to report during the past season: 1) if their child had been exposed to alcohol consumption at the club during junior matches, competition or junior training sessions; 2) if their child had been exposed to alcohol consumption at junior club events, such as junior presentation days, club barbeques (BBQs) and fundraisers, or junior registration days; 3) how often they (the parent) consumed alcohol during junior matches, competitions or training sessions; and 4) how often they (the parent) consumed alcohol at junior club events such as junior presentation days, club BBQs, and junior registration days.

##### Child exposure to tobacco

Parents of junior players were asked to report during the past season: 1) if their child had been exposed to tobacco smoke at the club during junior matches, competition or junior training sessions; 2) if their child had been exposed to tobacco smoke at junior club events, such as junior presentation days, club BBQ’s and fundraisers, or junior registration days; 3) how often they (the parent) smoked tobacco during junior matches, competitions or training sessions; and 4) how often they (the parent) smoked tobacco at junior club events such as junior presentation days, club BBQ’s, and junior registration days.

##### Child healthy food purchases

Parents were provided with a list of food items commonly available at sporting clubs [24], and asked which foods their child (or the parent on behalf of their child) usually purchased from the club canteen or shop. Categories of food items covered both healthy and unhealthy food options, and there was an ‘other’ option where parents could add any additional foods not already listed.

##### Child healthy drink purchases

Parents were provided with a list of beverages commonly available at junior sporting clubs [24], and asked which drinks their child (or the parent on behalf of their child) usually purchased from the club canteen or shop. Categories of beverages covered both healthy and unhealthy options, and there was an ‘other’ option where parents could add any additional drinks not already listed.

##### Club encouraged parents to provide healthy snacks for junior players

Parents of junior players were asked: “How does your club encourage you as a parent to provide healthy snacks for your child (ren) when they attend junior club games and events?” and then presented with the following options: 1) through club website and social media pages; 2) by sending letters, newsletters and emails to members; 3) at the venue (e.g. posters, signs or in registration packs); 4) verbal communication (e.g. ground announcements, by coaches); 5) other [please specify]; or 6) my club doesn’t do this.

##### Equal participation for children in training and games

Parents were asked to rate on a scale from 1 to 5 (from ‘strongly disagree’ to ‘strongly agree’) how much they agreed with the statement “My child spent as much time involved in training and on the field during games during this football season as other children in their team.”

##### Safe playing environment for children

Parents of junior players were asked to report whether they signed the club’s Code of Conduct policy in the past season.

Data for these secondary outcome measures were collected from parents of junior players via a CATI at baseline and post-intervention. The same parents who completed the survey at baseline were followed-up and invited to complete the survey post-intervention (cohort design). The survey items were developed by the research team and based on survey items used in previous trials in senior sporting clubs conducted by the authors.

##### Opportunities for regular physical activity for children

De-identified data regarding the number of junior players (under 18 years) registered to play at each club in the 2016 and 2017 seasons were obtained from each league to measure the impact of the intervention on opportunities for children to be physically active.

#### Demographic characteristics

Characteristics of the club (football code, number of players/teams, and geographic location), as well as the demographic characteristics of the club representative (gender, age, role at the club) were collected during the club representative CATI. The demographic characteristics of parents of junior players (gender, age, education level, income) were collected during the parent CATI. Demographic items were based on those used in the Australian Household National Survey [30].

#### Acceptability of the intervention

Nominated representatives from intervention clubs provided feedback regarding the usefulness of intervention components. Club representatives were asked: “What Good Sports Junior Resources did you find useful?” and “What Good Sports Junior resources did you not find useful?” Club representatives were presented with the following list of intervention components and asked to select all that applied: phone calls; social media message templates; reminder emails; action plans; posters; fact sheets; and checklists.

### Sample size and power

A sample size of 40 clubs per group at follow-up would enable the detection of a difference of 63% of a SD (or 0.63 units of z-score) between groups for all continuous outcomes reported by the club representative, with 80% power at the 0.05 significance level. A sample size of 200 parents per group from 80 clubs at follow-up, with an intra-class correlation (ICC) of 0.05, would yield an effective sample size of 139 parents per group (assuming an 80% response rate). Comparing139 parents per group would enable the detection of a reasonable difference in behaviour across secondary outcomes (with 80% power at the 0.05 significance level) including: a 15% increase in healthy food purchases by/for children (from 20 to 35%) [24] and a 17% increase in healthy drink purchases by/for children (from 50 to 67%) [24]. The ICC of 0.05 is a conservative estimate and is based upon previous ICC’s ranging from 0.01–0.05 used by the authors for related studies on alcohol reduction in sporting clubs [31] and healthy product purchasing from primary school canteens [32].

## Analyses

All analyses were performed in SAS 9.3. Descriptive statistics were used to describe the demographic characteristics of participating clubs, club representatives, and parents of junior players. The median number of junior players (under 18 years) registered to play at each club at baseline was used to categorise them as either a small club (< 169 registered players under 18 years) or a large club (≥ 169 registered players under 18 years). When categorising club representative roles, ‘Committee members’ included the club President, Vice-president, Secretary, Treasurer, or other club executive positions, while ‘Coach, Team Manager, other’ covered any non-executive positions. A multiple linear regression model was used to examine between group differences in the mean number of policies and practices implemented at the club level at follow-up. The model included a variable for group, where the control group was the reference, as well as a variable for baseline implementation (Yes/No) to adjust for baseline effect.

Secondary outcomes at the club level were analysed using multiple logistic regression models to examine between group differences in the proportion of clubs implementing each of the 16 policies and practices at follow-up. Secondary outcomes at the parent level were analysed using mixed effects logistic regression models to examine between group differences in the proportion of parents who reported their child had been exposed to a health promoting environment at the club at follow-up, where club ID was included as a random effect to account for potential clustering effect.

In all models, the control group was entered as the reference variable, and all models were adjusted for baseline values of policy and practice implementation (club representative reported data), or exposure to a health promoting environment (parent reported data). For two club practices no baseline data was available, therefore only post-intervention differences were analysed using all available data. For all other practices, analyses were conducted under an intention-to-treat framework using all available data. For missing data, multiple imputation was used via the MI procedure in SAS. The alpha value for significance testing was 0.05.

## Results

Of the 118 AFL and Rugby League football leagues that were identified in the states of NSW and Victoria, eight met the inclusion criteria for the study (See Fig. 1). These eight leagues were randomised to an intervention group (*n* = 4) and a control group (*n* = 4). Within these eight leagues, 89 eligible junior football clubs were identified, 79 of which agreed to participate in the trial (89%) and had a representative complete the telephone interview at baseline (Intervention group: 41; Control group: 38). Twenty-two clubs were lost to follow-up (*n* = 14 from the intervention group, *n* = 8 from the control group) with the majority (*n* = 18) unable to be contacted for post-intervention data collection. Twenty-seven intervention club representatives and 30 control club representatives completed the follow-up CATI.

Twenty out of 79 participating clubs did not provide any contact details for parents or carers of junior players. The remaining 59 clubs provided contact details for 387 parents and carers, and of these 336 (87%) were able to be contacted. From these, 141 parents in the intervention group, and 156 parents in the control group agreed to participate and completed the survey (total of *n* = 297, response rate of 79%). Excluding clubs that did not provide any parent or carer contact details, the average number of completed parent surveys per club was five.

The follow-up CATI was completed by 58 parents (out of *n* = 141, 41%) from 18 intervention group clubs and 74 parents (out of *n* = 156, 47%) from 27 control group clubs. There were no significant differences in the age, gender, education level or income of parents who completed the follow-up CATI, compared to parents who did not. All eight leagues that were initially randomised remained throughout the entire study.

### Demographic characteristics

Almost all intervention clubs were located in a metropolitan region, while around one fifth of control clubs were located in regional areas (18% at baseline, 20% at follow-up). There was a higher proportion of large clubs (169 members or more) in the control group at post-intervention, compared to the intervention group (83% vs 63% respectively). The club representatives who completed the CATI were usually committee members, and the majority of participating parents were female (65% or more), had a tertiary education (47% or more), and had an income of $1500 or more per week (40% or more) (Table 1).

**Table 1:** Characteristics of participating clubs, club representatives and parents at baseline and post-intervention

		Baseline		Post-intervention	
Characteristic		Intervention *n* (%)	Control *n* (%)	Intervention *n* (%)	Control *n* (%)
Clubs		*N* = 41	*N* = 38	*N* = 27	*N* = 30
*Football code*	AFL	23 (56)	20 (53)	16 (59)	18 (60)
	Rugby League	18 (44)	18 (47)	11 (41)	12 (40)
*Club size*^*a*^	Small (< 169 members)	21 (58)	13 (43)	10 (37)	5 (17)
	Large (≥169 members)	15 (42)	17 (57)	17 (63)	25 (83)
*Location*	Metropolitan	40 (98)	31 (82)	27 (100)	24 (80)
	Regional	1 (2.4)	7 (18)	0 (0)	6 (20)
Club representatives		*N* = 41	*N* = 38	*N* = 27	*N* = 30
*Age*	M (SD)	46.7 (7.3)	45.3 (6.2)	49.3 (8.4)	46.3 (6.1)
*Gender*	Male	20 (49)	21 (55)	17 (63)	20 (67)
	Female	21 (51)	17 (45)	10 (37)	10 (33)
*Role at club*	Committee member^b^	35 (85)	38 (100)	25 (93)	29 (97)
	Coach, Team manager, other	6 (15)	0 (0)	2 (7.4)	1 (3.3)
Parents		*N*= 141	*N*= 156	*N* = 57	*N* = 74
*Age*	M (SD)	42.7 (5.9)	43.2 (5.9)	44.7 (5.5)	44.7 (5.7)
*Gender*	Male	39 (28)	54 (35)	16 (28)	22 (30)
	Female	102 (72)	102 (65)	41 (72)	52 (70)
*Education level*	High School	31 (22)	28 (18)	13 (23)	11 (15)
	Trade/certificate	42 (30)	43 (28)	17 (30)	18 (24)
	Tertiary	68 (48)	85 (54)	27 (47)	45 (61)
*Weekly income*	<$800	41 (30)	40 (27)	12 (24)	20 (31)
	$800–$1499	37 (27)	48 (33)	16 (32)	17 (26)
	$1500	57 (42)	58 (40)	22 (44)	28 (43)

^a^168 players was the median club size and was as the cut point for categorising clubs as ‘small’ or ‘large’

^b^‘Committee members’ included the club President, Vice-president, Secretary, Treasurer, or other executive position

### Primary outcome: mean number of policies and practices implemented

No clubs reported implementing all 16 policies and practices at baseline, however one club reported implementing all 16 policies and practices at follow-up. The mean number of practices (out of 16) implemented by intervention clubs was 10.05 (SD = 1.50) at baseline, and 11.07 (SD = 1.59) at post-intervention. The mean number of practices implemented by control clubs was 9.89 (SD = 1.94) at baseline and 11.17 (SD = 2.25) at post-intervention. There was no significant difference between the intervention and control group regarding the mean number of practices implemented by clubs at post-intervention (Estimate − 0.05; 95% CI -0.91, 0.80; *p* = 0.90).

### Secondary outcome: proportion of clubs implementing each policy and practice

There were no significant differences between the proportion of intervention and control clubs implementing any of the practices at post-intervention (Table 2).

**Table 2:** Baseline and post-intervention data of reported club implementation of practices and child exposure to a health promoting environment

	Baseline		Post-intervention		Results following imputation		
Proportion of clubs that reported implementing each policy and practice	Intervention *N* = 41	Control^b^ *N* = 38	Intervention *N* = 27	Control^b^ *N* = 30	OR^c^	95% CI	*p*
	*n*(%)	*n*(%)	*n*(%)	*n*(%)			
Alcohol not available or consumed during junior competition	41 (100)	38 (100)	26 (96.3)	29 (96.7)	0.76	[0.04–13.64]	0.85
Alcohol not available or consumed at junior events or presentations	21 (51.2)	15 (39.5)	9 (33.3)	11 (36.7)	0.94	[0.32–2.78]	0.91
Alcohol not in change rooms when players under 18 yrs. are present	41 (100)	38 (100)	27 (100)	30 (100)	–	–	–
Alcohol manufacturers/retailers not advertised on any junior apparel	39 (95.1)	36 (94.7)	22 (81.5)	29 (96.7)	0.18	[0.02–1.67]	0.13
Alcohol not used for prizes, rewards or for fundraising	41 (100)	32 (84.2)	24 (88.9)	24 (80.0)	1.77	[0.40–7.81]	0.45
Club is compliant with the relevant state tobacco legislation	40 (97.6)	37 (97.4)	25 (92.6)	28 (93.3)	1.08	[0.14–8.47]	0.94
Club promotes all junior events as smoke free	26 (63.4)	26 (68.4)	18 (66.7)	16 (53.3)	1.82	[0.60–5.52]	0.29
Water is promoted as the drink of choice for junior players	39 (95.1)	36 (94.7)	22 (81.5)	25 (83.3)	1.14	[0.29–4.51]	0.85
Multiple healthy food and beverage options available at canteen/BBQ	25 (61.0)	22 (59.5)	18 (69.2)	21 (70.0)	0.85	[0.27–2.67]	0.78
Healthy options at canteen/BBQ promoted/displayed prominently	12 (29.3)	12 (32.4)	14 (53.9)	16 (53.3)	1.02	[0.37–2.83]	0.97
Club encourages parents to provide healthy snacks for junior players	41 (100)	38 (100)	24 (88.9)	29 (96.7)	0.33	[0.03–4.14]	0.39
At least one recruitment activity conducted to attract/retain players	^a^	^a^	16 (59.3)	19 (63.3)	0.85	[0.25–2.96]	0.80
Club has policy to ensure players get equal participation opportunity	^a^	^a^	23 (85.2)	21 (70.0)	2.51	[0.68–9.31]	0.17
Club has a Code of Conduct policy and records member agreement	31 (75.6)	30 (79.0)	19 (70.4)	24 (80.0)	0.60	[0.17–2.05]	0.41
Club has a Spectator Behaviour policy that is promoted and visible	14 (34.2)	14 (36.8)	11 (40.7)	12 (40.0)	1.06	[0.36–3.13]	0.91
Club has Good Sports Junior policy outlining health promoting practices	1 (2.4)	2 (5.3)	1 (3.7)	1 (3.3)	1.12	[0.07–18. 22]	0.94
Number of junior registered players at club: Mean (SD	183.28 (87.93)	254.52 (149.16)	210.96 (112.91)	333.52 (190.75)	–	–	0.08
Proportion of children reported to be exposed to a health promoting environment at club	Intervention *N* = 141	Control *N* = 156	Intervention *N* = 57	Control *N* = 74			
	*n*(%)	*n*(%)	*n*(%)	*n*(%)	OR	95% CI	*p*
Child not exposed to alcohol at club	55 (39.0)	63 (40.4)	20 (35.1)	29 (39.2)	1.16	[0.41–3.28]	0.78
Child not exposed to tobacco at club	93 (66.0)	118 (75.6)	22 (38.6)	28 (37.8)	0.97	[0.45–2.10]	0.94
Safe playing environment for child	80 (56.7)	95 (60.9)	49 (86.0)	66 (90.4)	0.81	[0.25–2.64]	0.72
Club encourages parents to bring healthy snacks	^a^	^a^	34 (59.7)	47 (63.5)	0.88	[0.34–2.31]	0.80
Child usually purchases healthy food at the club	15 (10.6)	12 (7.7)	4 (7.0)	10 (13.5)	0.49	[0.11–2.27]	0.35
Child usually purchases healthy food at the club	15 (10.6)	12 (7.7)	4 (7.0)	10 (13.5)	0.49	[0.11–2.27]	0.35
Child usually purchases healthy drinks at the club	68 (48.2)	60 (42.3)	29 (50.9)	30 (40.5)	1.48	[0.72–3.05]	0.27
Equal participation for children during training and games	^a^	^a^	54 (94.7)	71 (96.0)	0.76	[0.14–4.08]	0.74

^a^Only post-intervention data was collected for the practice

^b^The control group is the reference variable

^c^Odds Ratio of the intervention group versus the control group implementing the practice or policy at follow-up, adjusting for implementation at baseline

### Secondary outcome: child exposure to a health promoting environment at the club

At post-intervention, there were no significant differences in the proportion of children who were reported to be exposed to a health promoting environment at intervention clubs, compared to control clubs (Table 2).

### Acceptability of the intervention

All components of the intervention (phone calls; social media message templates; reminder emails; action plans; posters; fact sheets; and checklists) were reported to be useful by 95% intervention club representatives. When asked which components of the intervention were most useful, 53% reported reminder emails, with posters, phone calls and factsheets also chosen by more than one third of clubs.

## Discussion

To our knowledge, this pilot study is the first to test a multi-component implementation intervention in junior sporting clubs targeting a range of health risk behaviours. The trial found modest changes in trial outcomes following the intervention, none of which reached statistical significance. The findings highlight the challenges faced when attempting to support the implementation of heath promoting policies and practices in this setting.

The post-intervention outcomes were surprising given that the trial adopted some of the implementation support strategies and targeted similar policies and practices as those used in previous trials in adult community sporting clubs. For example, a previous cluster RCT targeted the availability, promotion and purchase of healthy food and beverage options in community football clubs [24]. At post-intervention, clubs who had been allocated to the intervention group were significantly more likely to have fruit and vegetable products available for purchase at the club canteen, significantly more likely promote fruit and vegetable products via reduced pricing and meal deals, and club members were significantly more likely to report purchasing fruit and vegetable products, compared to members of control clubs [24]. Similarly, significant improvements have been reported in the implementation of alcohol management practices and member alcohol consumption in previous randomized trials in community football clubs. Both interventions used a similar suite of implementation support strategies. The findings also contrast with analogous literature in settings such as schools where large improvements in health promoting environments have been achieved following implementation support in these settings [24, 33].

A number of factors may have contributed to the small and non-significant effects reported in this trial. First, the intervention may not have been long enough to enable change to occur at the club. Other multi-component intervention trials conducted in senior sporting clubs that have been successful in effecting change at both the club and club member level have been conducted over two or more sporting seasons [22–24]. Second, sporting clubs are dynamic environments, and are characterized by volunteer and transient staff who report considerable barriers to implementation of health initiatives. The inclusion of such a large and diverse number of policies and practices may have been too complex for clubs to execute. Implementation of a more targeted intervention, with a smaller number of policies and practices may have been more feasible for clubs to achieve.

Greater in-person support may have also strengthened the potential effectiveness of the intervention. There is evidence to suggest that face-to-face support may have an increased likelihood of motivating changes in behavior due to the ability to build stronger rapport, model and demonstrate practices, and gauge whether feedback and advice have been understood via non-verbal cues [34]. This is a strategy that was used in previously successful trials in senior sporting clubs [22, 24], and may have been a major contributor to their success. Other strategies that were used in these previous trials, such as observational audit and feedback, financial reimbursement, and peer-comparison feedback were not used in the current study [22], and may have also accounted for the non-significant change in junior sporting club practices.

Finally it appears that a number of practices (e.g., prohibiting alcohol from being present in change rooms when players under 18 years are present, prohibiting alcohol from being sold or consumed during junior competition, and clubs encouraging parents to provide healthy snacks for junior players) which formed the intervention criteria were already being implemented by a large proportion of clubs. Ceiling effects are present for such practices reducing the potential for further improvement. For future trials conducted with junior sporting clubs that meet Level 3 accreditation in the *Good Sports* program (such as those in the current trial), these practices could reasonably be removed from the intervention content given the already high level of adherence.

### Limitations

The interpretation of the findings presented should be considered within the limitations of the pilot study objectives and study methodology. All primary and secondary outcomes related to practices implemented by the club were assessed via self-report from club representatives. While this may have resulted in some social desirability bias, previous validation studies by the authors have shown that self-report by organisational representatives has high agreement with data collected via direct observation [35, 36]. There was also a high level of participant attrition, with 29% of clubs lost to follow-up, and 56% of parents lost to follow-up However, the loss was relatively equal across intervention and control groups, with most attrition attributed to being unable to contact representatives or parents, or conduct interviews at suitable times. The number of clubs who were eligible and participated (*n* = 41 intervention clubs versus *n* = 38 control clubs) was lower than the number estimated as needed in the sample size calculation. The smaller than anticipated sample and the high rate of attrition is likely to have impacted the power available to detect any significant differences. However, the effect size reported for trial outcomes were modest and typically far smaller than was hypothesised in the apriori sample size calculation. Finally, a large number of secondary outcomes were tested in the study, which may have had the potential to increase the risk of type 1 errors.

## Conclusions

This pilot study adds valuable information to the existing body of knowledge regarding the best ways to support junior sporting clubs to provide a healthier environment for their members. The findings suggest that the content of the intervention, and the strategies used to support implementation, require further refinement in order to be effective. Future studies should be better powered to detect significant findings, and questions regarding the acceptability of the intervention content and implementation strategies, as well as any potential barriers to implementation, should be included.

## Additional file

Additional file 1: Description of all survey questions used to assess the implementation of the 16 policies and practices. (DOCX 26 kb)

## Abbreviations

AFL: Australian Football League
BBQ: Barbeque
CATI: Computer Assisted Telephone Interview
NSW: New South Wales
