# Supplementary material for: Implementing health policies in Australian junior sports clubs: an RCT
Source: BMC Public Health. 2019 May 14;19:556. doi: 10.1186/s12889-019-6873-3 (PMC6515613; doi:10.1186/s12889-019-6873-3)
Supplement: Supplementary file 1 — Description of all survey questions used to assess the implementation of the 16 policies and practices. (DOCX 26 kb) [file 12889_2019_6873_MOESM1_ESM.docx]

| **16 Policies and Practices** | | **CATI Question** |
| --- | --- | --- |
| **Child alcohol exposure** | | |
| 1. | Alcohol is not available or consumed during junior competition | Club representatives were asked to answer ‘yes’ or ‘no’ to the following:   1. Does your club currently serve or allow alcohol to be consumed during junior matches or competitions at your home venue? This includes games and special events such as tournaments and gala days. |
| 2. | Alcohol is not available or consumed at junior events or presentations | Club representatives were asked to answer ‘yes’ or ‘no’ to the following:   1. Does your club currently serve or allow alcohol to be consumed during junior presentation events that your club holds at either your home? 2. Does your club currently serve or allow alcohol to be consumed during junior events such as fundraisers, club barbeques and registration days that your club holds at either your home venue or elsewhere? |
| 3. | Alcohol is not present in change rooms when players under 18 years are present | Club representatives were asked to answer ‘yes’ or ‘no’ to the following:   1. Does your club currently serve or allow alcohol to be consumed by adults in the club change rooms at your home venue during junior matches or competitions? |
| 4. | Alcohol manufacturers, wholesalers, retailers or other businesses whose core function is to sell alcohol are not promoted/advertised on any junior apparel | Club representatives were asked to answer ‘yes’ or ‘no’ to the following:   1. Does your club currently allow the display of branding or logos of alcohol manufacturers or distributors on junior players or coaches uniforms or apparel? This includes alcohol brands, alcohol retailers such as a liquor store, or alcohol manufacturers such as a winery. It does not include pubs. |
| 5. | Alcohol is not used for prizes, rewards or for fundraising | Club representatives were asked to answer ‘yes’ or ‘no’ to the following:   1. Is alcohol used as a prize or reward by the junior club? For example, as an end of season reward for a junior team coach or manager. If your club includes both seniors and juniors, we are only asking about prizes or rewards provided by the junior club. |
| **Child tobacco exposure** | | |
| 6. | The club is compliant with the relevant state tobacco legislation regarding smoke free areas during junior sporting events | Club representatives were asked to answer ‘yes’ or ‘no’ to one of the following questions that aligned to the specific tobacco legislation of their state (NSW or Victoria):   1. NSW - Are all areas of your sporting venue's spectator areas smoke-free during an organised sporting event? This includes any area set aside for, or being used by spectators to watch an organised sporting event. 2. Victoria - Has smoking been banned within 10 metres of your home venue ground during under 18s competitions, events and training sessions? Note this includes outdoor spectator seating as well as outdoor dining and drinking areas, and refers to any part of the venue used to conduct the sporting event. |
| 7. | The club promotes all junior events as smoke free | Club representatives were asked to answer ‘yes’ or ‘no’ to the following:   1. Does your club promote all junior events that your club holds at either your home venue or elsewhere, such as fundraisers, club barbeques and registration days as being completely smoke free environments? |
| **Child nutrition** | | |
| 8. | Water is promoted by the club as the drink of choice for junior players | Club representatives were asked to answer the following:   1. Does your club promote drinking water through any of the following strategies?   Club representatives were able to select more than one response from the following list:   1. Promotional signs or posters 2. Promotional announcements 3. Prominent positioning of products 4. Water included in meal deals 5. Free tap water provided 6. Reduced pricing 7. Other 8. Club does not promote drinking water |
| 9. | Multiple healthy food and beverage options (e.g. fruit, vegetables and non-sugar sweetened drink) are available for sale at junior club venue during games/events (canteen or barbeque) | Club representatives were asked to answer the following:   1. What types of food does your club sell? 2. And, Which of the following non-alcoholic drink options does your club sell or provide?     Club representatives were able to select more than one response from the following list for question 1):   1. Hot Chips, wedges or fried potatoes 2. Pies 3. Sausage Rolls 4. Sausage Sizzle 5. Hot dogs 6. Chiko Rolls 7. Confectionery lollies, sweets, eucalyptus lollies, 8. Chocolate Bars 9. Packet Chips, Twisties etc 10. Cakes, lamingtons, slices, doughnuts etc 11. Chocolate coated Ice-creams 12. Sandwiches (not salad) 13. Salad sandwiches 14. Fresh fruit (whole fruit) 15. Other fruit products (dried, frozen, canned) 16. Fruit salad 17. Salads 18. Fresh vegetables (whole veggies) 19. Vegetable salads 20. Other vegetable products (dried, frozen, canned) 21. Cheese & biscuits 22. Dried fruit 23. Steak sandwiches 24. Bacon & egg rolls 25. 99% fruit ice blocks 26. Popcorn 27. Rice wheels 28. Lean meat with added veg (eg savoury mince, burger with salad) 29. pasta, rice or noodles 30. Pizza 31. Other (please specify)   Club representatives were able to select more than one response from the following list for question 2):   1. Regular soft drinks (eg. coke, lemonade) 2. Diet soft drinks (eg. diet coke) 3. Juices (eg. fruit juice, apple juice) 4. Bottled/Mineral water 5. Free water 6. Plain milk 7. Flavoured milks 8. Sports drinks (eg. staminade, gatorade) 9. Hot drinks (eg. tea, coffee, hot choc) 10. Energy drinks 11. Other |
| 10. | The purchase of healthy choices at junior club games/events (canteen or barbeque) is promoted by ensuring healthy food and beverage options are displayed prominently | Club representatives were asked to answer the following:   1. Does your club promote ‘healthy’ food options such as fruit, vegies or salad sandwiches through any of the following strategies?   Club representatives were able to select more than one response from the following list:   1. Promotional signs or posters 2. Promotional announcements 3. Prominent positioning of products 4. Healthy food meal deals 5. Reduced pricing 6. Other 7. Club does not sell vegies/fruit/salad 8. Club does not promote ‘healthy’ food options |
| 11. | The club encourages parents to provide healthy snacks (e.g. fruit and water) for junior players | Club representatives were asked to answer ‘yes’ or ‘no’ to the following:   1. Does your club or coaches recommend fruit or water be provided to players after games or at half time?” |
| **Child participation in physical activity** | | |
| 12. | The club conducts at least one recruitment activity prior to the beginning of the sporting season to attract new junior players and retain current players | Club representatives were asked to answer ‘yes’ or ‘no’ to the following:   1. Does your club conduct any recruitment strategies to attract new players and retain current players?” |
| 13. | The club has a Participation policy that it communicates to members, coaches, officials and volunteers to ensure junior players are provided with equal opportunities for participation at both training and during games | Club representatives were asked to answer ‘yes’ or ‘no’ to the following:   1. Does your club currently have a Good Sports junior policy that includes information about equal participation for juniors during both training and games?   Club representatives were also asked to answer the following:   1. How does your club communicate its expectations of equal participation during junior games and training to members?   Club representatives were able to select more than one response from the following list for question 2):   1. Through club website and social media pages 2. By sending letters, newsletters and emails to members 3. At the venue (eg. posters, signs or registration packs) 4. Verbal communication (eg. ground announcements, coaches) 5. Other [please specify] 6. Club hasn't communicated this to members |
| 14. | The club has a Code of Conduct policy which it communicates to all members, and ensures member agreement is recorded | Club representatives were asked to answer ‘yes’ or ‘no’ to the following:   1. Does your club currently have a code of conduct policy for all members? 2. If yes, Is it a requirement that all members sign the code of conduct policy?   Club representatives were also asked to answer the following:   1. How does your club communicate its expectations of conduct and behaviour to members?   Club representatives were able to select more than one response from the following list for question 3):   1. Through club website and social media pages 2. By sending letters, newsletters and emails to members 3. At the venue (eg. posters, signs or registration packs) 4. Verbal communication (eg. ground announcements, coaches) 5. Other [please specify] 6. Club hasn't communicated this to members |
| 15. | The club has a Spectator Behaviour policy that is promoted and clearly visible at the club | Club representatives were asked to answer ‘yes’ or ‘no’ to the following:   1. Does your club currently have a separate spectator behaviour policy? 2. If yes, Is it a requirement that all parents or carers of junior members sign the spectator behaviour policy? |
| **All risk factors** | | |
| 16. | The club has a written Good Sports Junior policy, which outlines the club’s practices with regards to alcohol consumption, tobacco use, healthy eating and physical activity at junior competitions and events | Club representatives were asked to answer ‘yes’ or ‘no’ to the following:   1. Does your club have a written alcohol management policy? 2. If yes, Is your alcohol management policy Good Sports approved? 3. If yes, Does your club's alcohol management policy clearly state its alcohol management practices relevant for junior clubs or teams? 4. Does your club have a written smoke free policy? 5. If yes, Is your smoke free policy Good Sports approved? 6. If yes, Does your club's smoke free policy clearly indicate its smoke-free practices in regards to junior matches, competitions, or events? 7. Does your club have a written healthy eating policy? 8. If yes, Is your healthy eating policy Good Sports approved? 9. If yes, Does your club’s healthy eating policy clearly indicate its healthy eating practices in regards to junior matches, competitions, or events? 10. Does your club currently have a Good Sports junior policy that includes information about equal participation for juniors during both training and games? 11. Does your club currently have a code of conduct policy for all members? 12. If yes, Is it a requirement that all members sign the code of conduct policy? 13. Does your club currently have a separate spectator behaviour policy?” 14. If yes, Is it a requirement that all parents or carers of junior members sign the spectator behaviour policy? |
